# Supplementary material for: Effect of Free-Range and Low-Protein Concentrated Diets on Growth Performance, Carcass Traits, and Meat Composition of Iberian Pig
Source: Animals (Basel). 2020 Feb 11;10(2):273. doi: 10.3390/ani10020273 (PMC7070829; doi:10.3390/ani10020273)
Supplement: Supplementary file 1 [file animals-10-00273-s001.pdf]

animals

MDPI

Annex S1: Estimation of the intake of acorns and grass by Iberian pigs in extensive rearing system.

The estimation of the intake of acorns and grass by the pigs was carried out in three steps:

Firstly, we calculated the total metabolizable energy intake (ME intake) according to the regression equation described by Nieto et al. [19] (1), which relates the average daily gain (ADG) (g), the body weight (BW) (kg) and the feeding level, expressed as a multiple of energy requirements for maintenance ME_m, (ME intake:ME_m):

$$\mathrm{ADG} = -286 \pm 27 + 3.44 \pm 0.17 \times \mathrm{BW} + 185 \pm 8 \times \mathrm{ME\ intake{:}\ ME_m}$$
$$(\mathrm{n} = 99;\ \mathrm{r}^2 = 0.936;\ \mathrm{RSD} = 52.5) \qquad (1)$$

From this equation, ME intake was calculated to be 16.7 Mcal per pig and day, taking into account the following data: ADG = 1049 g/day (from FR pigs; see Table 1); BW = 145.4 kg (average BW of the period from FR pigs); ME_m = 98.4 kcal/kg $BW^{0.75}$ (according to Nieto et al. [19]); Coefficients: to choose the appropriate value of these coefficients within the range given, we adjusted the above equation (Eq. 1) with the data of LP and SP pigs, since from these two lots we knew all the values of ADG, BW, ME intake and ME_m. The equation was adjusted when these coefficients were at the highest value of the mentioned ranges: -259 (-286+27), 3.61 (3.44+0.17) and 193 (185+8).

Secondly, the estimated ME intake was divided between acorns and grass consumption according to the results of Rodríguez-Estévez et al. [20], who established that acorns and grass contribute 90.4 and 9.6%, respectively, of the total supply of energy to the grazing pig.

Thirdly, the consumption was calculated by taking 4.2 Mcal ME/kg of acorn kernel DM and 2.46 Mcal ME/kg of grass DM of energy contents [3].

So, finally, the estimated intake of acorn and grass consumed by FR pigs was 3600 g of acorn kernel DM and 655 g of grass DM per day.
